# Antibiotic prescribing patterns for management of acute diarrheal diseases at a university teaching hospital in Central Ethiopia

**DOI:** 10.1186/s40780-026-00592-0

**Published:** 2026-06-06

**Authors:** Mengistu Girma, Sisay Endale, Tamrat Assefa Tadesse

**Affiliations:** 1 Department of Pharmacy, College of Health Sciences, Wachemo University, Hossana, Ethiopia; 2 Department of Social and Administrative Pharmacy, School of Pharmacy, College of Health Sciences, Addis Ababa University, Addis Ababa, Ethiopia; 3 Department of Pharmacology and Clinical Pharmacy, School of Pharmacy, College of Health Sciences, Addis Ababa University, Addis Ababa, Ethiopia

**Keywords:** Antibiotic utilization, Acute diarrheal diseases, Nigist Elleni Mohammed Memorial Teaching Hospital

## Abstract

**Background:**

Antibiotic resistance has emerged largely due to the improper use of antibiotics in clinical practice, although most acute diarrheal diseases resolve without antibiotic treatment and should be reserved for cases of invasive bacterial or dysenteric diarrhea. This study aimed to assess the patterns of antibiotic use for acute diarrheal diseases at Nigist Elleni Mohammed Memorial Teaching Hospital (NEMMTH) in Central Ethiopia.

**Methods:**

A retrospective cross-sectional study was conducted to assess patients treated for diarrhea between January 1 and June 30, 2023. The appropriateness of antibiotic prescriptions for diarrhea was evaluated according to the Ethiopian Standard Treatment Guidelines. Data were collected using a structured abstraction form, and patient charts were selected through systematic random sampling. The analysis was performed using the Statistical Package for Social Sciences version 25.

**Results:**

Among 302 patients evaluated, 53.3% were female and 46.4% were children under five years old. Of the 302 patients, 57.6% had watery diarrhea. A total of 74.5% of patients received at least one antibiotic, of whom 73.5% received a single antibiotic during the treatment of acute diarrheal cases. The most commonly prescribed antibiotics were metronidazole (18.2%), cotrimoxazole (17.5%), and ciprofloxacin (11.6%). The proportion of inappropriate antibiotic prescribing was 77.1%.

**Conclusions:**

This study found a high level of inappropriate antibiotic use for acute diarrheal disease at NEMMTH. Metronidazole and cotrimoxazole are the most commonly prescribed antibiotics. Ensuring proper management by adhering to the standard treatment guidelines is crucial.

## Introduction

Diarrhea is the leading cause of mortality in children worldwide. It is the third leading cause of death in children under five years of age and is responsible for killing approximately 443,832 children every year. Globally, there are nearly 1.7 billion cases of childhood diarrheal disease every year. Diarrhea can last for several days and leave the body without water or salts that are necessary for survival [1]. Diarrhea can be categorized as acute or chronic, according to its duration. Acute diarrhea, characterized by the passage of three or more watery stools for a maximum of 14 days, is a common cause of mortality in developing countries. It is more frequently caused by viral infections, which typically present as mild, non-inflammatory, and self-limiting [2, 3]. Regardless of the cause or degree of disease severity, rehydration therapy is the cornerstone of supportive treatment and should be combined with appropriate dietary support [4, 5].

Empirical antibiotic therapy is advised because bacteria are not detectable in more than 90% of instances of diarrhea. However, when evaluating the clinical efficacy of empirical antibiotic therapy, the possibility of side effects and the elimination of beneficial microbes should be considered [6, 7]. Antibiotics are crucial for treating bacterial infections, but are often misused. They are recommended for serious cases of acute diarrheal diseases such as bloody diarrhea or dysentery. However, research has shown that inappropriate use is common, leading to antibiotic resistance, which is a significant threat to public health [8].

Antibiotic resistance is prevalent in community diseases, and inappropriate use of antibiotics contributes to 20–50% of all antibiotic use [9, 10]. Furthermore, most hospitals in developing countries have improper antibiotic utilization rates [11]. Viral and self-limiting infections, such as severe diarrhea, are the most common causes of antibiotic abuse. Viruses such as rotavirus account for 70% to 75% of all cases of diarrhea [12]. Several studies have revealed different forms of antibiotic misuse in hospital settings in developed and developing nations, which leads to unnecessary costs for bacterial infections and an increase in antibiotic resistance [13–17]. Antibiotic utilization patterns are crucial for implementing antibiotic stewardship programs (ASP) in healthcare settings [18–20]. Assessing community use of antibiotics for acute diarrhea is essential to guide interventions that promote rational use, reduce costs, and curb AMR. The findings of this study provide evidence to strengthen antibiotic stewardship in this setting. This study at Nigist Elleni Mohammed Memorial Teaching Hospital (NEMMTH) aims to determine the prevalence and types of antibiotics used in managing acute diarrheal illnesses.

## Methods

### Study setting and period

The study was conducted at NEMMTH, located in Hosanna Town, Hadiya Zone, Central Ethiopia, approximately 230 km south of Addis Ababa, Ethiopia. It provides preventive, curative, and rehabilitative clinical services organized into many case teams. This study was conducted from January 1, 2023, to June 30, 2023.

### Study design

A retrospective cross-sectional study was conducted to evaluate antibiotic use patterns for acute diarrheal illnesses at the NEMMTH.

### Source and study population

The source population comprised all patients with diarrhea at NEMMTH, whereas outpatients diagnosed with acute diarrheal disease and treated for it from January 1, 2023, to June 30, 2023, were considered the study population.

### Sample size determination and sampling techniques

The sample size for the study was calculated using a single-population proportion formula targeting a 95% confidence interval with an estimated prevalence rate of 50% and a 5% margin of error. Initially, 384 participants were required; however, this was reduced to 287 because of the population of fewer than 10,000 participants. An additional 5% was added to address potential data issues, resulting in a final sample size of 302. A systematic random sampling technique was used to select the patient charts. The sampling interval (k = 4) was established by dividing the total number of charts by the sample size and selecting every 4th chart. The first chart was chosen randomly from the first 4 charts based on the record order.

### Data collection instrument and procedures

Data were collected using structured data abstraction formats. The acquired information included patients’ sociodemographic and clinical features, antibiotic prescriptions on charts, and prescription profiles. We also collected and classified stool characteristics based on the laboratory reports. All tests performed were stool examinations by microscopy. When multiple symptoms were recorded, only one chief complaint was assigned per patient using a predefined classification rule that prioritized the primary symptom documented at presentation (or the symptom most directly related to the main reason for the visit and clinical diagnosis/treatment decisions), thereby avoiding duplication in the analysis.

### Outcome measurement

In this study, diarrhea was diagnosed based on World Health Organization criteria, defined as the passage of three or more loose or watery stools within a 24-hour period. The study evaluated the appropriateness of antibiotic prescriptions for diarrhea management based on the recommendations provided in the Ethiopian Standard Treatment Guidelines (STG) [21]. The STG outlines the recommended first-line antibiotic treatments, including the specific medication, dosages, and durations, for different types of diarrheal illnesses. Furthermore, these guidelines recommend antibiotics only for patients with bloody diarrhea, for a duration of more than 7 days, or in the presence of specific signs of invasive disease. The assessment of appropriateness was solely based on the documented diagnosis and prescribed medications recorded in the patients’ medical records.

### Data processing and analysis

After checking for completeness and consistency, the data were entered into Statistical Package for Social Sciences version 25. Descriptive statistics, such as frequency and percentage, were calculated, and the data were presented using narratives, tables, and figures.

### Ethical considerations

Ethical clearance was obtained from the Research Ethics Committee of the College of Medicine and Health Sciences of Wachemo University (CMHS/Phar.D-1998/2022). Informed consent was deemed unnecessary for this study, as no patient interviews were conducted and data were collected retrospectively from medical charts with authorization granted by the NEMMTH Medical Director’s Office.

## Results

### Socio-demographic characteristics of the patients

Of the 302 patients included in the study, 161 (53.3%) were female. Children under five years comprised 140 (46.4%) patients, while those aged 19–35 years accounted for 75 patients (24.8%) (Table 1).

**Table 1:** Socio-demographic characteristics of patients with acute diarrheal disease (*n* = 302)

Characteristics	Category	Frequency	Percentage (%)
Sex	Male	141	46.7
	Female	161	53.3
Age (in years)	< 5	140	46.4
	5–18	70	23.2
	19–35	75	24.8
	36–50	10	3.3
	51–65	4	1.3
	> 65	3	1

### Clinical characteristics of the patients

Fever (33.4%), fever and vomiting (21.5%), and vomiting (15.9%) were the common symptoms documented in patients with acute diarrheal diseases. More than half of the patients (56%) had experienced the illness for two to three days, with a mean of 2.02 (SD ± 0.71) days. All patients underwent stool examination, and 57.6% had watery stools, followed by bloody stools (22.8%) (Table 2).

**Table 2:** Clinical characteristics of patients with acute diarrheal diseases at NEMMTH (*N* = 302)

Characteristics		*N* (%)
Common symptoms	Fever	101(33.4)
	Fever and vomiting	65 (21.5)
	Vomiting	48 (15.9)
	Abdominal cramp	46 (15.2)
	Nausea	42 (13.9)
Illness duration	Less than one day	67 (22.2)
	Two to three days	169 (56)
	Four to seven days	60 (19.9)
	More than seven days	6 (2)
Stool characteristics	Watery diarrhea	174 (57.6)
	Bloody diarrhea	69 (22.8)
	Mucoid diarrhea	59 (19.5)

### Treatment patterns of acute diarrheal diseases

Of the total 302 patients with acute diarrhea, approximately three-fourths (255, 74.5%) were prescribed antibiotics. All (100%) patients with bloody diarrhea, 59.8% of patients with watery diarrhea, and 88.1% of patients with mucoid diarrhea received antibiotics (Table 3).

**Table 3:** Treatment pattern of acute diarrheal diseases treatment at NEMMTH

Antibiotics prescribed	Watery (*N* = 174)		Bloody (*N* = 69)		Mucoid (*N* = 59)	
	*n*	%	*n*	%	*N*	%
Yes (*N* = 225)	104	59.8	69	100	52	88.1
No (*N* = 77)	70	40.2	0	0	7	11.9
Total	174	100	69	100	59	100

Two hundred twenty-two (73.5%) patients received one antibiotic during the treatment of acute diarrheal cases (Metronidazole was the most commonly prescribed drug (18.2%), followed by cotrimoxazole (17.5%) (Table 4).

**Table 4:** Antibiotics used for acute diarrheal diseases treatment at NEMMTH (*N* = 302)

Prescribed antibiotics description		Frequency	Percentage
Number of antibiotics prescribed	No antibiotics	36	15.3
	One antibiotics	222	73.5
	Two antibiotics	34	11.2
Prescribed antibiotics	Metronidazole	55	18.2
	Cotrimoxazole	53	17.5
	Azithromycin	36	11.9
	Ciprofloxacin	35	11.6
	Amoxicillin + Clavulanic Acid	30	9.9
	Cephalexin	13	4.3
	Metronidazole +Ciprofloxacin	14	4.6
	Ciprofloxacin + Doxycycline	10	3.3
	Ciprofloxacin + Cotrimoxazole	6	2.0
	Metronidazole + Cotrimoxazole	4	1.3

Note: The medications were administered orally or by injection, depending on patient status and the available preparations

### Adherence to standard treatment guidelines

The appropriateness of the antibiotic prescription pattern was determined by regularly assessing adherence to STG. In our study, 233 (77.1%) patients were not treated in accordance with the STG, and 156 (51.6%) were prescribed antibiotics for non-bloody diarrhea.

### Prescribers’ profile for management of acute diarrheal diseases

Nearly half of the patients with acute diarrhea (50.3%) were treated by medical interns, while 34.4% of the cases were treated by general practitioners. In 23 instances (7.6%), the prescribers were not identified. Specialists showed higher adherence to the national STG for antibiotic prescriptions, with a compliance rate of 65.2% (Table 5).

**Table 5:** Prescriber profile on acute diarrheal diseases at NEMMTH

Prescribers	Prescribed in line with STG		Not prescribed in line with STG	
	Frequency	Percentage	Frequency	Percentage
Specialist (*n* = 23)	15	65.2	8	34.8
General practitioner (*n* = 104)	57	54.8	47	45.2
Medical intern (*n* = 152)	73	48.1	79	51.9
Unknown (*n* = 23)	11	47.8	12	52.2
Total	142	47.0	160	53.0

## Discussion

This institution-based retrospective cross-sectional study was conducted to evaluate the pattern of antibiotic use for acute diarrheal diseases in NEMMTH, Hossana, Central Ethiopia. In this study, 74.5% of the patients who received treatment for acute diarrhea had taken at least one antibiotic. This result is significantly lower than that reported in studies conducted in India, Ethiopia, and West India, where 71%, 84%, and 75.1% obtained an antibiotic prescription for an acute diarrheal disease, respectively [22–24]. The World Health Organization (WHO) guidelines for drug use indicators recommend the percentage of encounters with antibiotics to be within the range of 20-26.2% [25]. The current study results are higher than those of similar studies carried out in various nations, which indicated that the percentage of encounters with antibiotics was between 9.1 and 52.3%, which is lower than the results reported in the current study [26–30]. Patient pressure on physicians for antibiotic prescription may be an influencing factor for prescribers [31].

A key factor in developing countries is the high prevalence of infectious diseases, which leads to increased antibiotic use and widespread empirical treatments. In addition, healthcare professionals may inconsistently follow treatment guidelines. The findings indicated that most patients with acute diarrheal disease experienced symptoms such as vomiting and fever for two to three days, suggesting viral infection. Although 174 patients (57.6%) had watery diarrhea, which typically does not require antibiotics, approximately 104 patients (60%) were treated improperly with antibiotics. This irrational use contributes to antibiotic resistance, as most cases of community-acquired diarrhea are viral, and antibiotics do not halt disease progression but can disrupt normal gut flora [27]. High levels of inappropriate antibiotic use for acute diarrheal disease at the NEMMTH may result from limited awareness of the guidelines, insufficient educational opportunities, and a lack of communication among healthcare professionals. These factors underscore the need for targeted training interventions, and stakeholders should provide training to healthcare providers on the rational use of antibiotics and adherence to the STGs to ensure appropriate management of acute diarrheal diseases and help prevent the spread of antimicrobial resistance.

From the total reviewed patient charts of acute diarrheal diseases, approximately 69 (22.85%) cases had clinical features of bloody diarrheal disease that required antibiotic use, and all of them were prescribed antibiotics, which is in line with the antibiotic use recommendations for diarrheal disease [32]. On the other hand, the percentage of acute diarrheal patients treated not in line with STG was 51.6%, which is slightly better than that of a study conducted in Bishoftu, Ethiopia (72.3%) [28]. These results are comparable to those reported in China 51.3%) [29] and Thailand (49%) [24]. A study from South Thailand showed that 73.8% of antibiotics prescribed were in line with STG for diarrheal disease treatment [33].

In this study, metronidazole (18.2%) and cotrimoxazole (17.5%) were the most commonly prescribed antibiotics for acute diarrhea. Similar patterns have been reported in studies from Thailand and Ethiopia, where cotrimoxazole was the most commonly used agent, followed by amoxicillin and ciprofloxacin [24, 34]. This may be explained by the fact that these agents are among the most familiar empiric choices for presumed infectious diarrhea in routine clinical practice in Ethiopia. Cotrimoxazole, ciprofloxacin, and levofloxacin have been the first-line choices for most bacterial infections [35], although there is an alarming increase in resistance to these antibiotics [36–37]. The study also showed that children under five years of age were the most affected, and most antibiotics were prescribed to this age group. However, these age groups require particular attention when it comes to antibiotic use, and antibiotics are inappropriately administered.

In this study, interns accounted for the highest proportion of prescriptions (50.3%) and exhibited the highest rate of nonadherence to treatment guidelines (51.9%). This pattern may be attributed to several structural and experiential factors, including limited clinical experience, incomplete familiarity with standard treatment guidelines, limited exposure to institutional prescribing principles, and reliance on empirical decision-making, all of which have been shown to increase deviations from the guidelines among junior prescribers [38–40]. Inadequate and inconsistent supervision by senior clinicians, coupled with brief orientation and limited hands-on training in antibiotic prescribing, may further contribute to suboptimal prescribing practices. Additionally, easy access to antibiotics, even in the absence of clear clinical indications, may encourage liberal prescribing. These practices contrast with the recommended standards that emphasize structured prescribing education, close supervision, and regular audits and feedback. Strengthening targeted training programs, supervision mechanisms, and guideline-based prescribing support may reduce inappropriate prescribing by interns managing acute diarrheal diseases.

The current study had some limitations. This study was conducted at a single referral hospital in Central Ethiopia, which limits the generalizability of its findings to other settings. Its retrospective design, based on chart review, may also be affected by incomplete or inaccurate documentation. Another limitation was that when multiple symptoms were documented, the chief complaint was selected based on the predefined classification rule, which may have introduced misclassification or selection bias and could affect the accuracy of symptom categorization. This study is also limited by the use of a pre-existing dataset with restricted variables and the absence of detailed patient-level information (e.g., comorbidities, disease severity, and clinical outcomes), which constrained additional analyses, limited the assessment of prescribing appropriateness, and highlighted the need for future prospective studies with more comprehensive data collection.

## Conclusions

The study found high levels of inappropriate antibiotic use for acute diarrheal disease at the NEMMTH. Metronidazole and cotrimoxazole are the most commonly prescribed antibiotics. Children under five years of age were the most affected, with most prescriptions deemed inappropriate. Stakeholders should monitor management practices and ensure adherence to STG, while providing training on the rational use of antibiotics and antibiotic resistance.

## Data Availability

The datasets used in this study are available from the corresponding author upon request.

## Abbreviations

AMR: Antimicrobial Resistance
ASP: Antimicrobial stewardship program
NEMMTH: Nigist Eleni Mohammed Memorial Teaching Hospital
STGs: Standard treatment guidelines
WHO: World Health Organization
